# Arming the oncolytic virus enadenotucirev (EnAd) to generate enhanced locally-acting immunotherapies for cancer

**DOI:** 10.1186/2051-1426-3-S2-P334

**Published:** 2015-11-04

**Authors:** Brian R Champion, Nalini Rasiah, Sam Illingworth, Hugo Calderon, Matthieu Besneux, Rochelle Lear, Darren Plumb, Prithvi Kodialbail, Alice Brown

**Affiliations:** 1 PsiOxus Therapeutics Ltd, Oxford, UK

## Background

EnAd is a potent, chimeric Ad11p/Ad3 adenovirus with selective oncolytic activity against a range of epithelial cancer cells. Ongoing clinical studies have shown that i.v. dosed EnAd infects and selectively replicates in tumor cells, producing detectable levels of viral protein, often with an associated high frequency of intra-tumoral CD8^+^ cells. These data indicate that transgene-encoded proteins will also be effectively produced within tumors using an “armed” EnAd virus.

## Methods

We are therefore developing EnAd viruses that are armed for selective delivery of immunotherapeutics to tumors following systemic dosing. EnAd is highly human-tumor selective and does not replicate, produce infectious progeny or express endogenously regulated transgenes in non-human cells. Preclinical evaluation of immuno-modulatory activities of armed viruses is therefore a major challenge and requires application of multiple approaches that can collectively provide informative data.

To evaluate local immunomodulatory effects of EnAd viruses we have been working with the following systems:

1. *In vitro* co-cultures of primary human immune cells and tumor cells

2. *In vivo* studies in immunocompetent mice using i.t. dosing of mouse CT26 tumors with armed EnAd variants, where transgene expression is driven by a CMV promoter

3. *In vivo* studies with human tumor xenografts in immunodeficient mice with a reconstituted immune system, where armed EnAd viruses can be dosed systemically to selectively infect tumors and modulate local immune responses - in the context of viral infection and tumor cell lysis

## Results

Using these approaches we have shown that EnAd treatment results in sustained local production of transgenes in tumors and can also lead to maturation and activation of dendritic cells, upregulating CD80, CD86 and MHC class II following the oncolytic release of virus particles. This innate stimulation results in enhanced T cell activation as evidenced by increased tumor antigen-specific T cell responses and CD69 upregulation, IFN-γ & IL-2 secretion. For example, using a virus encoding the tumor associated antigen NY-ESO-1 (NG-217) injected into CT26 tumours we demonstrated the induction of antigen-specific T cell ELISPOT responses to both encoded NY-ESO-1 epitopes and CT26 tumor antigens. This correlated with retardation in tumor growth in NG-217 treated animals. Thus transgene expression and the presence of viral particles in the tumor can drive active anti-tumor T cell responses.

## Conclusions

These systems are now being used to evaluate efficacy and mechanism of action of immunotherapeutic agents, such as immune activators and checkpoint inhibitors, produced locally within tumor tissues following delivery of armed EnAd viruses.

